# A Comparative Evaluation of Arginine Complex Combined With Flouride and Two Standard Non-Fluoridated Remineralizing Agents: An In Vitro Study

**DOI:** 10.7759/cureus.60118

**Published:** 2024-05-11

**Authors:** Saurabh Joshi, Nilesh Vaidya, Bharti Gupta, Bhushan Pustake, Gaurav Shinde, Shilpa Pharande

**Affiliations:** 1 Department of Pediatric Dentistry, Rural Dental College, Pravara Institute of Medical Sciences, Loni, IND; 2 Department of Conservative Dentistry and Endodontics, Endodontics School of Dental Sciences, Krishna Vishwa Vidyapeeth, Karad, IND; 3 Department of Maxillofacial Surgery and Diagnostic Sciences, College of Dentistry, Jazan University, Jazan, SAU; 4 Department of Pedodontics and Preventive Dentistry, Mahatma Gandhi Vidyamandir’s Karmaveer Bhausaheb Hiray (KBH) Dental College, Nashik, IND; 5 Department of Pedodontics and Preventive Dentistry, Rajesh Ramdasji Kambe (RRK) Dental College and Hospital, Akola, IND; 6 Department of Orthodontics and Dentofacial Orthopaedics, Sinhgad Dental College and Hospital, Pune, IND

**Keywords:** bioactive glass, remineralization, non flouridated agents, diagnodent laser flourescence, arginine bicarbonate

## Abstract

Background

Dental caries represents a dynamic process, often reversible in its early stages. Fluoride has conventionally served as the cornerstone for remineralization and early caries arrest. However, excessive fluoride intake can lead to both local and systemic toxicity. Hence, there's a pressing need to develop adjunct therapies that enhance fluoride's efficacy while minimizing its dosage. This study aims to assess and compare the remineralization potential of a novel combination comprising arginine bicarbonate and fluoride against established technologies such as Bioactive glass (NovaMin Technology; Sensodyne Repair and Protect, GlaxoSmithKline, UK) and CPP-ACP technology (GC Tooth Mousse; Tokyo Japan).

Materials and methods

The experiment utilized extracted premolars designated for orthodontic extraction. The initial evaluation employed the DIAGNOdent^TM^ fluorescence method. Subsequently, teeth underwent demineralization and were measured for values. Following this, the teeth were subjected to seven cycles of remineralization, after which moment values were reassessed. Statistical analysis was performed on the recorded values.

Results

Participants were divided into six groups (BR-A, AR-A, BR-B, AR-B, BR-C, AR-C). T-tests demonstrated significant reductions in moment values within each group, indicating the effectiveness of all remineralizing agents. Group C exhibited the most substantial difference (-6.900 ± 0.4), followed by Group A and Group B. ANOVA analysis revealed statistically significant differences among all three groups (p=0.016). Tables showed significant distinctions between the remineralizing values of Groups A and C and Groups B and C (p=0.02 and 0.002, respectively), with no discernible distinction between Groups A and B.

Conclusion

The study elucidates the superior efficacy of the arginine complex with fluoride combination compared to CPP-ACP and Bioactive Glass individually. This finding underscores the potential of the novel combination therapy in enhancing remineralization while minimizing fluoride dosage, thus presenting a promising strategy for addressing early-stage dental caries.

## Introduction

Dental caries is one of the most common reasons for tooth loss in children. It is a dynamic process that can be arrested as well as reversed at a very early stage [1]. Since ancient times, more emphasis has been given to prevention rather than the reversal of an early carious lesion. Therefore, much of the population is still at a preventive stage. To understand the concept of a remineralizing agent, we must first understand the demineralization-remineralization cycle. The surface of the enamel is partially a by-product of the continuous process of demineralization and remineralization [2]. Enamel becomes demineralized when the pH of the biofilm drops below 5.5, resulting in the loss of calcium and phosphate and leading to the formation of a subsurface white lesion. This process is reversible provided a higher or neutral pH can be achieved [2,3]. The natural way of neutralizing the demineralization process is through the buffering action of saliva, which contains calcium and phosphate ions that return to the demineralized surface. There are currently various strategies in play to aid remineralization [3]. The main aim of all these agents is to directly deliver the lost ions onto the demineralized surface. Many materials have evolved with varied mechanisms of action [3].

Fluoride or fluoride-based materials have been established as one of the best remineralizing agents over the years. Fazzi et al. (1997) proved that fluoride permanently binds to enamel and forms fluorapatite, which creates a more stable crystal and is, therefore, more impervious to acid attacks [4]. However, high amounts of fluoride can cause local as well as systemic toxicity and thus add complications. Therefore, non-fluoridated agents have been developed since 1993, with the very first one being Casein Phospho-peptide Stabilized Amorphous Calcium Phosphate (CPP-ACP) technology, also known as RECALDENT by Dr. Eric Reynolds, Australia. ACP technology was introduced in 1996 [5]. CPP is a milk protein that can stabilize clusters of ACP into CPP-ACP molecules. At a neutral pH, CPP is a highly charged complex that can bind to various cation complexes around the tooth and increase the pH of the biofilm [5]. Then, ACP binds to the tooth surface, and slow release of calcium takes place, thus remineralizing the necessary surface. Enamelon was introduced by Dr. Tung. Sodium calcium phosphosilicate (Bioactive glass) can provide calcium, sodium, and phosphate ions to form Hydroxyl Carbonate Apatite (HCA) and release ions for remineralization [6]. A toothpaste named NovaMin was introduced by Dr. Len Litowsky and Dr. Gary Hack based on this formulation. Recent research regarding this technology was brought forth by Moradian in 2001 [6].

Quite recently, a new additive to fluoride has been recognized. Arginine is a semi-essential amino acid found in several salivary proteins and peptides. It is metabolized by the arginine deaminase pathway to produce energy, ammonia, and carbon dioxide by bacteria such as *Streptococcus sanguinis* [7,8]. The generation of ammonia increases the pH and neutralizes the acidic environment of the biofilm [8]. Arginine was initially recognized by Kleinberg in 1979 as a factor that increased oral pH. Clinical studies have proven its efficiency against cariogens time and again since 2000 [7]. It also has an additive effect on the remineralization potential of fluoride. In a study by Acevedo et al., it was seen that 1.5% arginine proved to be a successful adjunct to fluoride in dentifrice formulations [9]. Such a formulation was introduced by Ortek Therapeutics, named the CaviStat technology, incorporated initially in Denclude and then Proclude Dentifrices, Ortek Therapeutics [9,10]. It comprises arginine bicarbonate and particles of calcium carbonate, a common abrasive in toothpaste. The arginine complex adheres to the calcium carbonate on the enamel or dentin surface, allowing for the slow release of calcium molecules, thus promoting remineralization. This formulation is currently available as Colgate Sensitive Plus and Colgate Palmolive [11]. Therefore, this study aims to compare and evaluate the remineralization potential of the combination of arginine bicarbonate and fluoride with Bioactive glass (NovaMin) and CPP-ACP technology (GC Tooth Mousse).

While our study has provided compelling results, it is important to acknowledge its limitations. These may include the relatively small sample size, the short duration of the study period, and the specific methodology employed. Future research endeavors should aim to address these limitations by conducting larger-scale studies over longer durations, utilizing more diverse populations, and employing advanced analytical techniques to further elucidate the mechanisms underlying the observed effects of arginine complexes with fluoride on dental remineralization.

## Materials and methods

The study was conducted in the Department of Pediatric Dentistry, Rural Dental College, after obtaining clearance from the Institutional Ethical Committee, Pravara Institute of Medical Sciences (PIMS), Loni, with the approval number PMT/PIMS/IEC/2021/560. The materials required for the study are mentioned in Table 1.

**Table 1:** List of materials required for the study DIAGNOdent^TM ^(Kavo, Biberach, Germany)

Extracted teeth
10%bthymol
DIAGNOdent^TM^ pen with type B probe
Demineralizing agents
GC Tooth Mousse, Tokyo, Japan; CPP-ACP Technology (casein phosphopeptide stabilized amorphous calcium phosphate)
Sensodyne Repair and Protect, GlaxoSmithKline, UK; NovaMin technology (calcium sodium phosphosilicate)
Colgate Sensitive Plus, Pro Argin, Colgate-Palmolive, US; arginine bicarbonate complex combined with fluoride

**Figure 1:**
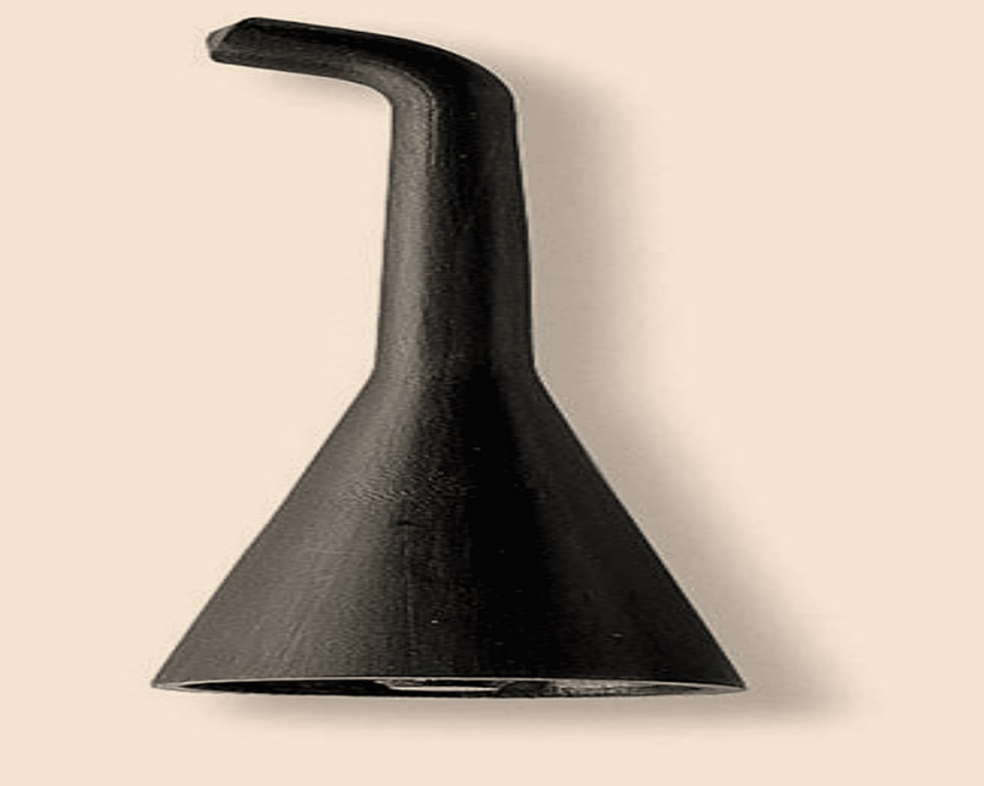
DIAGNOdent Type B probe DIAGNOdent^TM^ (Kavo, Biberach, Germany).

A total of 45 permanent extracted teeth were selected for the study. Caries-free premolars extracted for orthodontic purposes were used. Teeth with detectable or white spot lesions were excluded. The teeth were stored in 10% thymol after extraction. The teeth were cleaned of any calculus or debris. Each extracted tooth was coated in nail varnish, leaving an enamel window of 3 mm x 3 mm on the buccal surface of the tooth. Different colored varnishes were used for ease of sampling. Examinations of baseline readings were done using DIAGNOdent^TM^ (Kavo, Biberach, Germany). In our study, a DIAGNOdent^TM^ type B light probe with a flat surface was used (Figure 1). AS recommended by the manufacturer, prior to every measurement session, the instrument was calibrated against its own ceramic standards. 

Samples that showed a moment value between 3 and 7 were chosen. All the teeth with values above 7 were discarded. Finally, the teeth were divided into three groups of fifteen each as follows: Group A: Remineralization with GC Tooth Mousse, Tokyo, Japan; CPP-ACP technology (casein phosphopeptide stabilized amorphous calcium phosphate) (15 teeth), Group B: Remineralization with Sensodyne Repair and Protect, GlaxoSmithKline, UK; NovaMin technology (calcium sodium phosphosilicate) (15 teeth), Group C: Remineralization with Colgate Sensitive Plus, Pro Argin, Colgate-Palmolive, US; arginine bicarbonate complex combined with flouride (15 teeth).

The teeth were immersed in a glass container containing the demineralizing agent for 48 h at 37°C using an incubator (OSWARLD™, Mumbai, India). The demineralizing agent was composed of the following [12,13]; 2.2 mM monosodium phosphate, 2.2 mM calcium chloride, 0.05 M lactic acid, the final pH was adjusted to 4.5 using 50% sodium hydroxide using pH meter. After incubation, the teeth were washed with deionized water, dried with the help of an air syringe, and placed in three glass containers. The teeth were then evaluated again after one demineralization cycle using DIAGNOdent. Scanning electron microscopy (SEM) could have been used for the surface topography and energy dispersive X-ray spectroscopy (EDAX) for assessing the mineral content. Since the previous studies have shown results with no statistically significant difference for DIAGNOdent, SEM, and EDAX, hence DIGNOdent was the choice for our study. The teeth all showed moment values of over 9, which proved the existence of a white spot lesion. The samples were then treated with the respective remineralizing agents for seven days. The values were measured again using DIAGNOdent and the values were tabulated.

Statistical analysis

ANOVA (Analysis of Variance) was used for intergroup comparison, and the Student's unpaired 't' test was used for intragroup comparison. P Values of 0.05 were considered significant. All the collected data was tabulated and the master chart thus made was subjected to descriptive statistics such as mean and standard deviation in the Microsoft Excel Sheet, Version 2019. Analysis of Variance (ANOVA) was used to check intergroup and Student's Unpaired 't'-test was used for intragroup comparison. The graphs were prepared using GraphPad Prism software, Version 9.0.0 (GraphPad Software, Inc., Boston, MA).

## Results

**Figure 2:**
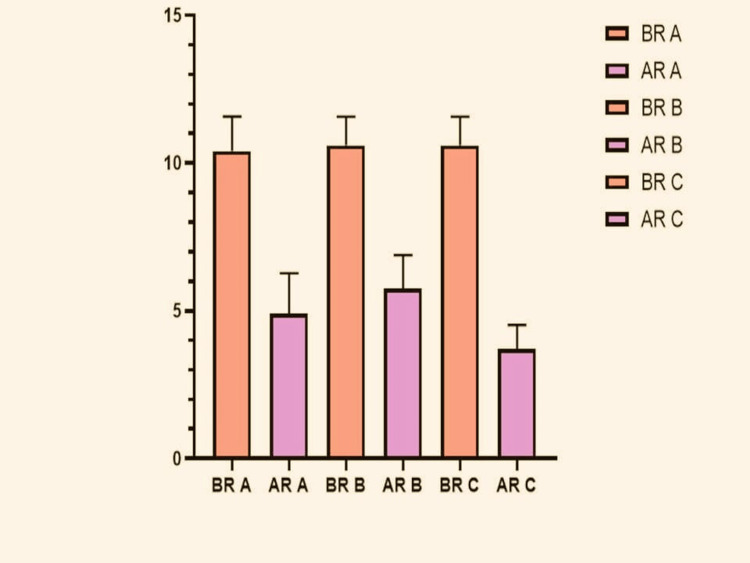
Mean and standard deviation values BR A: Before Remineralization Arginine; AR A: After Remineralization Arginine; BR B: Before Remineralization, Bioactive Glass; AR B: After Remineralization, Bioactive Glass; BR C: Before Remineralization Casein Phospho-peptide Stabilized Amorphous Calcium Phosphate (CPP-ACP); AR C: After Remineralization CPP-ACP.

For ease of analysis, the three groups were further divided into six groups: Before Remineralization Group A (BR-A), After Remineralization Group A (AR-A), and so on with Group B (BR-B and AR-B) and with Group C (BR-C and AR-C). The mean and Standard Deviation Values of each of the group's Pre and Post Remineralization are as seen in the graph below (Figure 2).

From the conducted ‘t’-tests the following observations were made. There was a significant decrease in moment values seen in each of the three intragroup comparisons which showed that all the used remineralizing agents proved effective. The maximum difference (Mean Difference Value: -6.900 ± 0.4) was seen with Group C, Followed by Group A and then Group B (Table 2). The ANOVA test was conducted for intergroup comparison between all three groups and the p-value obtained was statistically significant at 0.016

**Table 2:** Difference in remineralization values after using GC tooth mousse, Sensodyne repair and protect, Colgate Sensitive Plus: Pro Argin P value-Pearson's coefficient GC tooth mousse (Tokyo, Japan), Sensodyne repair and protect (GlaxoSmithKline, UK), Colgate Sensitive Plus: Pro Argin (Colgate-Palmolive, US).

Categories	Group A(GC Tooth Mousse)	Group B (Sensodyne Repair and Protect)	Group C (Colgate Sensitive Plus;ProArgin)
Before Remineralization	10.4±1.7	10.6±1.6	10.3±1.3
After Remineralization	4.9±1.3	5.7±1.7	3.7±0.86
Mean difference	-5.500 ± 0.5706	-4.850 ± 0.4717	-6.900 ± 0.4014
P Value	<0.0001	<0.0001	<0.0001

The results from the analysis, as presented in Table 3, provide valuable insights into the differences in remineralizing values among the three groups (Groups A, B, and C). When comparing Group A and Group C, a statistically significant difference in remineralizing values is observed, with a p-value of 0.02. This indicates that the treatments or conditions associated with these groups have a notable impact on remineralization, with Group C exhibiting significantly different remineralizing values compared to Group A. Similarly, when comparing Group B and Group C, the analysis reveals an even more pronounced difference in remineralizing values, with a p-value of 0.002. This suggests that the treatments or conditions represented by Group B are significantly different from those in Group C in terms of their effect on remineralization. The lower p-value indicates a stronger level of statistical significance, further supporting the conclusion of a substantial difference between these two groups. However, in the comparison between Group A and Group B, no statistically significant difference in remineralizing values is detected. This finding suggests that the treatments or conditions associated with these two groups do not result in significantly different remineralization outcomes when directly compared. The p-value for this comparison likely exceeded the predetermined threshold for statistical significance, indicating that any observed differences between Group A and Group B could have occurred due to random chance rather than a meaningful distinction in treatment effectiveness.

**Table 3:** Intergroup comparison between Group A, Group B and Group C after remineralization

Groups	Mean Values	P value
Group A & Group C	4.9±1.3	0.02
Group B & Group C	3.7±0.86	0.002
Group A & Group B	5.7±1.7	0.14

In summary, the results highlight the importance of considering the specific treatments or conditions implemented in each group when evaluating their impact on remineralization. While significant differences are observed between Group C and both Group A and Group B, indicating distinct treatment effects, no such difference is detected between Group A and Group B. These findings underscore the complexity of remineralization processes and emphasize the need for further research to elucidate the underlying mechanisms driving these observed differences.

## Discussion

In the above analysis, we have compared the remineralizing potential of three technologies: arginine bicarbonate with fluoride, casein phosphopeptide stabilized amorphous calcium phosphate, and bioactive glass (calcium sodium phosphosilicate). Evaluation of values was done at baseline, before and after remineralization (Table 4).

**Table 4:** Baseline values of teeth before demineralization

Group	Group A	Group B	Group C
Mean values	0.0	0.0	0.0

In vitro evaluation of demineralization and remineralization can be done using several techniques such as SEM/Environmental Scanning Electron Microscope (E-SEM), DIAGNOdent, surface microhardness, etc. [12,13]. Various authors have used either one or more of these methods to confirm their analysis. In our study, we have used the DIAGNOdent method to assess the difference between pre- and post-remineralization.

DIAGNOdent laser fluorescence is a method used to measure early demineralization of the teeth. It is based on the principle that when a DIODE laser of 655 nm wavelength is irradiated on the dental surface, the enamel and dentin absorb and reflect red fluorescence. Greater moment values detect greater lesions. In our study, the teeth used were initially demineralized using a demineralizing agent. It was incubated for 48 hours and then put through seven cycles of remineralization. The values were measured at baseline, after demineralization, and post-remineralization. The method used was following studies conducted by Bahrololoomi et al. [13] and Joshi et al. [12].

In a study conducted by Schupbach et al., the anticariogenicity of CPP-ACP was observed and the microbial content of saliva was reduced [14]. In another analysis conducted by Oshiro et al., it could be seen that CPP-ACP paste application had a positive effect on the remineralization of tooth surfaces [15]. In another previous assessment conducted by Mehta et al. in 2014, it was proven that bioactive glass had a greater surface attachment and, consequently, greater microhardness compared to CPP-ACP which was disproven by our study [16]. According to our study, CPP-ACP proved to have more remineralization potential than bioactive glass. In an earlier study conducted by Guclu et al. in 2017, it was seen that when they compared the remineralization potential of CPP-ACP, bioactive glass, and hydroxyapatite (HAP) crystals, they found HAP to be superior to both of the other remineralizing agents, and CPP-ACP was found to better than bioactive glass [17]. This was found to be per our study results as well.

The success of fluoride as a remineralizing agent has been proven repeatedly over the years by various authors such as Ten Cate JM [18], Roveri et al. [19], and Gao et al. [20]. Fluoride has also been much superior compared to other non-fluoridated remineralizing agents, especially while facing acid challenges, and it has been proven by authors such as Oliviera et al., whose in vitro analysis proved that 1.1% NaF was a better remineralizing agent as compared to 10% CPP-ACP [21]. Bioactive glass (NovaMin) technology has proven to be at least as effective, if not better than fluoride. However, in an acid challenge, a high amount of fluoride has proven to be the best agent for remineralization. Unfortunately, increasing the fluoride content in most preparations would result in the adverse effects of fluoride toxicity. Therefore, an effective adjunct would be ideal as it would promote more fluoride uptake in lesser quantities of fluoride as well. This makes it safe even for the pediatric age group.

Arginine has been discovered as an agent increasing the pH of the biofilm since the 1980s. Since then, various studies conducted have proved the efficiency of arginine bicarbonate complex in combination with fluoride in a dentifrice formulation. In an experimental analysis conducted by Ten Cate and Cummins, 1.5% arginine bicarbonate complex and fluoride in a dentifrice formulation increased the reversal and arrest of early carious lesions in children [22]. In another assessment done by Bijle et al., it was proven that the formulation of 2% arginine and NaF showed a strong antimicrobial effect on biofilms containing *S. mutans* [23]. In an analysis conducted by Cheng et al., it became clear that the addition of arginine improved fluoride uptake and, therefore, the overall remineralization potential of the combined complex [24]. The results of the aforementioned studies were based on our results as well. Our study proved that the arginine bicarbonate complex combined with fluoride proved to be more effective than CPP-ACP as well as the bioactive glass technology.

The drawbacks of the present in vitro study include the non-evaluation of subsurface remineralization, although surface remineralization was confirmed. Invitro remineralization is quite different from in vivo remineralization in the oral cavity. Thus, the creation of clinical conditions must exercised with caution because of the obvious limitations of in vitro studies.

## Conclusions

Very few studies have been conducted to prove the efficiency of arginine complexes for their remineralizing potential. With the limitations of our study, the present study proved arginine complexes with fluoride as superior to non-fluoridated remineralizing agents. Arginine complexes showed the highest remineralizing potential, followed by GC tooth mousse and followed by NovaMin technology. However, more studies with larger samples are required to come to conclusive results.
